# Microdystrophin Expression as a Surrogate Endpoint for Duchenne Muscular Dystrophy Clinical Trials

**DOI:** 10.1089/hum.2022.190

**Published:** 2023-05-17

**Authors:** Jeffrey S. Chamberlain, Melissa Robb, Serge Braun, Kristy J. Brown, Olivier Danos, Annie Ganot, Pedro Gonzalez-Alegre, Nina Hunter, Craig McDonald, Carl Morris, Mark Tobolowsky, Kathryn R. Wagner, Olivia Ziolkowski, Dongsheng Duan

**Affiliations:** ^1^ Department of Neurology, Sen. Paul D. Wellstone Muscular Dystrophy Specialized Research Center, University of Washington, Seattle, Washington, USA; ^2^ Robb Consulting (contractor of REGENXBIO, Inc.), Columbia, Maryland, USA; ^3^ AFMTelethon, Evry, France; ^4^ Solid Biosciences, Inc., Charlestown, Massachusetts, USA; ^5^ REGENXBIO, Inc., Rockville, Maryland, USA; ^6^ Spark Therapeutics, Inc., Philadelphia, Pennsylvania, USA; ^7^ University of California Davis Medical Center, Sacramento, California, USA; ^8^ Hyman, Phelps & McNamara, P.C. (former contractor of REGENXBIO, Inc.), Washington, District of Columbia, USA; ^9^ F. Hoffmann-La Roche, Ltd., Basel, Switzerland; ^10^ Former contractor of REGENXBIO, Inc., Rockville, Maryland, USA; and; ^11^ Department of Molecular Microbiology and Immunology, Neurology, Biomedical Sciences, and Biomedical, Biological & Chemical Engineering, University of Missouri, Columbia, Missouri, USA.

**Keywords:** microdystrophin, Duchenne muscular dystrophy, gene therapy, AAV vector

## Abstract

Duchenne muscular dystrophy (DMD) is a serious, rare genetic disease, affecting primarily boys. It is caused by mutations in the *DMD* gene and is characterized by progressive muscle degeneration that results in loss of function and early death due to respiratory and/or cardiac failure. Although limited treatment options are available, some for only small subsets of the patient population, DMD remains a disease with large unmet medical needs. The adeno-associated virus (AAV) vector is the leading gene delivery system for addressing genetic neuromuscular diseases. Since the gene encoding the full-length dystrophin protein exceeds the packaging capacity of a single AAV vector, gene replacement therapy based on AAV-delivery of shortened, yet, functional microdystrophin genes has emerged as a promising treatment. This article seeks to explain the rationale for use of the accelerated approval pathway to advance AAV microdystrophin gene therapy for DMD. Specifically, we provide support for the use of microdystrophin expression as a surrogate endpoint that could be used in clinical trials to support accelerated approval.

## DUCHENNE MUSCULAR DYSTROPHY

Duchenne Muscular Dystrophy (DMD) is a recessive, X-linked neuromuscular disorder caused by mutations in the *dystrophin* gene, which spans ∼2.4 megabases and includes 79 exons.^1,2^ These mutations lead to little or no dystrophin production (typically <3% of the normal quantity of dystrophin).^3^ Dystrophin is a protein critical in physically stabilizing the membranes of muscle cells. The near-total absence of full-length dystrophin production in DMD patients results in progressive muscle degeneration that manifests primarily as muscle weakness impairing walking, other motor functions, breathing, and cardiac function,^4^ with the most common cause of death being cardiorespiratory failure.^5^ Although the pace of symptom progression is heterogenous in DMD patients, muscle weakness typically begins between ages 3 and 5 years, with loss of ambulation usually occurring in the early teenage years and loss of ability to self-feed by the late teenage years.

Studies have reported a mean age at mortality ranging from 18.1 to 31.4 years.^6^ DMD predominantly affects males, with an estimated incidence of about 16 live male births per 100,000 in the United States.^7^ Rarely, females are affected by DMD, with around 8% of female carriers having some degree of muscle weakness or cardiomyopathy.^8^

## CURRENT LANDSCAPE OF DMD THERAPY

The U.S. Food and Drug Administration (FDA) has approved five treatments for DMD: deflazacort, eteplirsen, golodirsen, viltolarsen, and casimersen. Deflazacort is a glucocorticoid that was granted traditional approval, while the rest are exon-skipping drug products that were granted accelerated approval based on a mean increase in internally truncated dystrophin production in skeletal muscle (quantified using western blot [WB]). In 2018, FDA issued a final guidance to the industry on DMD drug development.^9^ The guidance notes that “FDA has no defined set of required or recommended clinical outcome measures for studies in dystrophinopathies…” and suggests that existing or novel outcome measures that can measure clinically meaningful effects may be appropriate.

Several types of medical products are currently under development for DMD. One direct way of treating this disease would be to restore the expression of dystrophin. Gene therapy based on adeno-associated virus (AAV)-mediated delivery of microdystrophin genes has emerged as a promising method since the gene encoding the full-length dystrophin protein is too large to fit inside a single AAV vector.^10^ Microdystrophin genes are designed to be small enough to fit into an AAV vector while retaining the primary functionality of the full-length dystrophin protein by being rationally designed to include the most critical protein domains. AAV vectors can transduce cells that are not actively dividing, and they are minimally integrating, nonpathogenic, and less immunogenic than gene therapies that use other delivery mechanisms.^11^

Trials are now studying the safety and efficacy of systemically administered AAV vectors to deliver different forms of microdystrophins to slow or stabilize the loss of muscle function throughout the body.^12^ We note that not all groups have adopted the expression “microdystrophin,” and in particular the Pfizer AAV trial for DMD uses what they call a “minidystrophin.” For simplicity, here, we use the term “microdystrophin” to describe any cDNA less than ∼4 kb that encodes a shortened dystrophin protein. This terminology distinguishes these AAV-sized dystrophins from larger “minidystrophins” that are encoded by Becker muscular dystrophy (BMD) patients carrying large deletions, that allow expression of coding regions in the 5–8 kb range.

## FDA'S ACCELERATED APPROVAL PATHWAY

The accelerated approval provisions allow the FDA to grant accelerated approval to “…a product for a serious or life-threatening disease or condition…upon a determination that the product has an effect on a surrogate endpoint that is reasonably likely to predict clinical benefit, or on a clinical endpoint that can be measured earlier than irreversible morbidity or mortality, that is reasonably likely to predict an effect on irreversible morbidity or mortality or other clinical benefit, taking into account the severity, rarity, or prevalence of the condition and the availability or lack of alternative treatments.”^13^

All products granted accelerated approval are subject to certain requirements, which may include postapproval studies to verify and describe clinical benefit.^14^ The importance of postapproval studies being conducted with due diligence, as required by the law and regulations,^15^ is paramount to the success of the accelerated approval program. Sponsors must ensure that the required postapproval studies are well designed and completed in a timely manner.

DMD is clearly a serious condition and AAV gene therapies could provide a meaningful advantage over available therapy, consisting of the regular use of steroids. This includes deflazacort, a corticosteroid that reduces inflammation and activity of the immune system and improves muscle strength in DMD patients.^16^ FDA may also consider prednisone, a therapy with off-label use in DMD, to constitute available therapy based on recommendations by authoritative scientific bodies that evaluate clinical evidence and other reliable information that reflects current clinical practice or standard of care.^17^ The ongoing use of steroids carries with it the risk of serious complications such as altered endocrine function, immunosuppression, and an increased risk of infection, osteoporosis, altered cardiovascular/renal function, and behavioral and mood disturbances.^18^

Meaningful advantages over available therapy could be based on effects that target the underlying cause of DMD, effects that are superior or additive to those of steroid therapy, or which provide treatment options to patients unable to tolerate or who are unresponsive to steroid therapies. It is worth noting that FDA does not consider products approved under the accelerated approval pathway based on a surrogate endpoint or intermediate clinical endpoint and where the clinical benefit has not been verified by postapproval studies as “available therapy.” This would include the four exon-skipping drugs approved for DMD.

The accelerated approval pathway is defined using an endpoint, either a surrogate or an intermediate clinical endpoint, which is reasonably likely to predict clinical benefit. A surrogate endpoint used as the basis for accelerated approval is not one that has been validated to show clinical benefit. Validated surrogate endpoints are known to predict clinical benefit and can be used for traditional approval.

FDA noted in its Final Rule on the Accelerated Approval Regulations that “…[w]hether a given endpoint is, in fact, reasonably likely to predict clinical benefit is inevitably a matter of judgment. FDA, using available internal and external expertise, will have to make informed judgments in each case presented, just as it does now. The agency acknowledges that there are well-recognized reasons for caution when surrogate endpoints are relied on… A sponsor must persuasively support the reasonableness of the proposed surrogate as a predictor and show how the benefits of treatment will outweigh the risks. Such presentations are likely to be persuasive only when the disease to be treated is particularly severe (so that considerable risk is acceptable) and/or when the surrogate endpoint is well supported. In addition, it will be the sponsor's clear obligation to resolve any doubts as to clinical value by carrying out definitive studies.”^19^

This article will describe the evidence supporting microdystrophin expression as a likely predictor of clinical benefit from AAV DMD gene therapy. Specifically, this article will discuss and provide support for the use of expression of microdystrophin in muscle as a surrogate endpoint reasonably likely to predict changes of muscle function that reflect a clinical benefit for DMD patients. This article advocates for the use of the accelerated approval pathway for approval of AAV gene therapies for the treatment of DMD.

## MICRODYSTROPHIN OVERVIEW

As mentioned previously, it is not possible to administer the full-length dystrophin coding sequence in an AAV vector to treat DMD because its size (∼11.5 kb) greatly exceeds the AAV packaging capacity (∼5 kb). This problem was addressed with the development of microdystrophin genes that are <4 kb. Microdystrophins are shortened but are functional versions of dystrophin.^12^ These microgenes include genetic sequences that have been curated to include elements identified as most critical for dystrophin function. Various gene therapy candidates aim to improve the DMD phenotype by delivering vectors expressing microdystrophin genes, which will produce minimized microdystrophin proteins that retain the key function of the full-length dystrophin protein by linking the subsarcolemmal cytoskeleton with the extracellular matrix and by recruiting primary members of the dystrophin-associated protein complex (DAPC) to stabilize the muscle.

The rationale is that the production of microdystrophin is reasonably likely to result in improved muscle function. FDA recognized that the production of larger internally truncated dystrophins was reasonably likely to predict clinical benefit for the four exon-skipping drugs granted accelerated approval for the treatment of DMD.

## BIOLOGICAL PLAUSIBILITY

The FDA has repeatedly asserted in its summary reviews of the four exon-skipping drugs granted accelerated approval that the role of dystrophin is well-characterized in the pathophysiology of DMD.^20–23^ Dystrophin is a critical muscle protein that is reduced or absent in DMD patients. Dystrophin's critical role is linking the subsarcolemmal actin cytoskeleton to the extracellular matrix via the DAPC.^24,25^ This link helps reduce muscle cell stress generated during contraction, while its absence leads to muscle cell injury, subsequent degeneration, and a gradual loss of muscle cells.^26^

Microdystrophins are shorter than full-length dystrophin normally produced endogenously, yet, they remain functional. Microdystrophin is considered therapeutic when the expression is durable and the protein is appropriately membrane-localized, recruits members of the DAPC, and stabilizes or increases muscle force generation, resulting in increased muscle strength and prevention of muscle cell necrosis.^27–30^

Protein size alone does not always correlate with the clinical phenotype. In fact, some individuals with shorter dystrophin (due to large deletions involving multiple exons) have milder diseases compared with those with longer dystrophin.^31^ In addition, disease severity of dystrophinopathy is determined not by the size of the gene but rather by the quantity, as well as the quality, of the dystrophin protein produced. Specifically, patients with severe disease have <5% of the normal quantity of dystrophin, whereas patients with dystrophin levels between 5% and 10% of normal, regardless of protein size, usually have an intermediate phenotype (mild DMD or severe BMD). In contrast, based on findings reported in a review article, patients with mild to moderate BMD phenotype usually have protein levels above 20%.^32^

## REGULATORY HISTORY OF SHORTENED DYSTROPHIN AS AN ENDPOINT FOR ACCELERATED APPROVAL

FDA has accepted a statistically significant increase in a shortened version of dystrophin as a surrogate endpoint supporting accelerated approval of four exon-skipping products thus far. For each of these four accelerated approvals, FDA relied upon demonstration of a small increase in *de novo* production of the internally truncated dystrophin protein in skeletal muscle. The four exon-skipping products showed a mean change from baseline (% normal dystrophin) of 0.28–5.3% (0.28% for eteplirsen, 0.92% for golodirsen, 5.3% for viltolarsen, and of 0.8% for casimersen).^20–23^

Although there was public disagreement about whether the magnitude of expression was meaningful, the FDA ultimately decided that increased dystrophin production in skeletal muscle was reasonably likely to predict clinical benefit and therefore was an acceptable surrogate endpoint for accelerated approval. Thus, the production of a shortened version of dystrophin resulting from treatment is established as an FDA-accepted surrogate endpoint to support accelerated approval of a product intended to treat DMD.

## RATIONALE FOR USE OF MICRODYSTROPHIN EXPRESSION AS A SURROGATE ENDPOINT

In addition to the regulatory precedent and biological plausibility described above, the use of microdystrophin as a surrogate endpoint provides a variety of valuable benefits. Unlike some functional endpoints currently used in trials, microdystrophin—as an objectively measured endpoint—reduces potential biases that can impact the results of clinical outcome assessments, a challenge FDA has identified in guidance regarding the use of these endpoints, and variables in patient motivation.^9,33,34^ In addition, the use of this surrogate endpoint allows a reduction in study duration. Clinical studies have reported that protein expression has been seen as early as 2 months after treatment with increased expression at 12 months, supported also by previous preclinical work.^30,35–39^ Functional outcomes have generally been assessed at 12 months in DMD studies. Therefore, the use of this surrogate endpoint could significantly shorten the time needed for demonstration of efficacy in DMD studies.

In addition, because all microdystrophin constructs used in clinical evaluation have been designed to produce controlled and consistent protein expression across muscle cells, the similarities between microdystrophins are closer than the variations induced by mutations in exon-skipped internally truncated dystrophin. For example, the eteplirsen approval was based on six different mutations amenable to exon-51-skipping therapy, although additional mutations are known to exist.

FDA noted that there may be some differences in functionality of the protein produced from exon-51-skipped transcripts, and that the different internally truncated dystrophins produced in patients with different mutations could also confound interpretation of possible effects on clinical course based on differences in dystrophin levels.^40,41^ As an example, while exon skipping typically removes a single exon from the transcript, each spectrin-like repeat (SR) in the dystrophin rod domain is encoded by about two exons. Thus, in many cases when an exon is skipped, the encoded protein carries only part of one of the SRs, which can impact the stability of the resulting internally truncated protein.^29,42^ More stable proteins result from maintaining properly phased SRs, which has become a gold-standard design strategy to produce the synthetic microdystrophins.^29^

## RATIONAL DESIGN OF FUNCTIONAL MICRODYSTROPHIN

The rational design and development of functional microdystrophins have been based on naturally occurring mutations in BMD patients and refined by repeated studies in DMD animal models, in which nonfunctional microdystrophin constructs are readily distinguished from functional versions through the durability of expression and improved functional outcomes.^28–30,43^

## PRECLINICAL STUDIES

Published studies have consistently shown that restoring dystrophin in muscles improves muscle morphology, strength, and resistance to contraction-induced injury. This was initially shown in 1993 by Cox et al, who expressed a full-length dystrophin coding sequence in striated muscles of transgenic *mdx* mice.^44^ In treated mice, expression levels up to 50 times normal restored DAPC localization, muscle histology, and strength without any associated toxicity. This proof-of-concept study revealed that gene therapy had the potential to treat DMD.

Similar results were obtained in 1993 and 1995 by different groups. First, minimized dystrophin was delivered by adenovirus-mediated transfer to *mdx* mice intramuscularly. In this study, expression of the truncated protein protected the fibers efficiently against the muscle degeneration process.^45^ Subsequently, two groups generated additional transgenic *mdx* mice expressing either full-length or internally truncated dystrophin, the latter of which was based on a BMD patient with a 1 MB deletion (of exons 17–48) who remained ambulatory until his death in his late 70s.^31^ Very mild phenotypes have been associated with deletion of exons 13–48, an even larger deletion.^46^ In these studies, dystrophic pathology was eliminated in muscles that expressed ≥20% of normal levels of full-length dystrophin, and higher levels of uniform expression did not further increase strength beyond normal.^47,48^

Internally truncated dystrophins patterned from BMD patients did not fully restore strength as did the full-length dystrophin, however, they completely halted ongoing necrosis and degeneration and led to largely normal muscle histology.^49^ These results provided the basis to develop even smaller microdystrophins with normal SR repeat phasing that could be administered using AAV vectors.

The first studies of functional microdystrophins small enough to be carried by AAV vectors were published starting in 2000. These microdystrophins are encoded by cDNAs smaller than ∼4 kb.^29,50^ In one study, three different microdystrophins were expressed in *mdx* mice by intramuscular injection of AAV vectors. All three of these vectors produced microdystrophins, although in different amounts, and improved muscle histology. Since delivery was localized, no measurements of strength were performed.^50^ In the second study, a variety of full-length dystrophins and microdystrophins were compared in transgenic *mdx* mice, and three different microdystrophins were further analyzed after intramuscular injection of the AAV vectors *mdx* mice.^29^ As in earlier studies, the larger dystrophins were often, but not always, better at restoring normal strength, although all constructs showed a significant benefit.^29^

With microdystrophins, maximally functional activity came from designs that preserved SR phasing and that favored the use of subdomains normally adjacent to each other in the native protein. These smaller proteins also retained the major protein-interaction domains within dystrophin and were assessed in multiple muscles and at various ages of the dystrophic mice. The critical elements identified in these studies are all incorporated into the design of the microdystrophins currently in clinical development. Finally, an earlier study tested IM injection of vectors carrying between one and three SR domains, but these constructs had minimal functional benefit leading to the conclusion that a minimum of four SRs is needed for significant functional benefit.^29,50–52^

Together, these various studies highlighted both the striking functionality of small dystrophins and that expression up to normal levels improved or eliminated dystrophy. In addition, these studies were the basis for identifying critical domains needed for functional microdystrophin constructs.

Subsequent studies moved toward testing systemic delivery of AAV vectors expressing various microdystrophin in striated muscles of *mdx* mice, and later, the canine DMD model. These studies showed the ability of microdystrophin in adult mammals to prevent and reverse pathology and increase strength.^53^ The degree of phenotypic improvement was also shown to depend on the dose of AAV vector delivered (and hence the amount of microdystrophin produced). Early systemic AAV delivery studies revealed the ability to deliver microdystrophin gene constructs to all striated muscles in a dose-dependent manner.^36,54^ Lower doses led to dystrophin expression in a mosaic pattern, which partially improved histology and strength, whereas higher doses led to more uniform levels of dystrophin and a more complete rescue of the dystrophic phenotype in mice.^36,55^ Phenotypic reversal was also observed in old *mdx* mice (up to 2 years old).^30,56–58^ Microdystrophin has been similarly effective in canine DMD models.^35,38,59–62^

## CRITICAL ELEMENTS OF FUNCTIONAL MICRODYSTROPHIN

**Figure 1.**
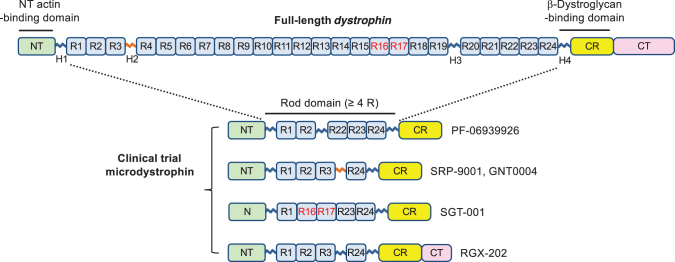
Critical elements of functional microdystrophin. Full-length *dystrophin* contains an NT domain (NT, amino acids 14–240), a central rod domain (amino acids 253–3040) with 24 spectrin-like repeats (R1 to R24) and four hinges (H1 to H4), a CR domain (CR, amino acids 3080–3360), and a CT domain (CT, amino acids 3361–3685). Functional microdystrophins all carry the NT actin-binding domain, CR/beta-dystroglycan-binding domain, and shortened rod domain with at least four spectrin-like repeats. Note, the amino acid number is based on the Leiden Muscular Dystrophy pages (https://www.dmd.nl). H2 is highlighted in red because it contains a polyproline site which profoundly influences the functional capacity of microdystrophin in murine studies.^85^ R16/17 is highlighted in red because these two repeats anchor neuronal nitric oxide synthase (nNOS) to the muscle cell membrane and protect muscle from functional ischemia in exercise.^43^ CT, C-terminal; CR, cysteine-rich; NT, N-terminal.

The ability to express functional microdystrophins from coding sequences small enough to be carried by AAV vectors is based on (1) studies of deletions that removed various domains from dystrophin and their effects on functionality of the protein, as discussed under Biological Plausibility, and (2) the knowledge gained from natural deletions occurring in BMD patients.^63^ The critical functional elements of dystrophin are described below (Fig. 1):

1. N-terminal actin-binding domain (ABD)
Truncations of the N-terminal ABD (N-ABD) have not resulted in favorable outcomes; consequently, the entire ABD is retained in all microdystrophins constructs. The primary reason is that such truncations greatly reduce the stability of dystrophin.^64–69^ Dystrophin also has a central ABD,^70–72^ and as long as the N-ABD is present and functional, the central ABD can be deleted.^28,64,70,73,74^
2. Cysteine-rich/beta-dystroglycan-binding domain (Dbd)
The beta-Dbd is composed of a WW domain within hinge 4 and the cysteine-rich domain.^63,75–78^ Essentially all mutations or deletions in this Dbd have been observed to inactivate dystrophin completely; hence this region is present in all functional microdystrophin constructs.^42,79–82^
3. Rod domain with hinge 1, hinge 4, and at least four SRs
Shortened dystrophins lacking all SRs are nonfunctional.^29^ Hinge1, hinge 4, and at least four SRs are needed to prevent dystrophy and increase strength in *mdx* mice.^29,30,43,50–52,83,84^ All microdystrophin constructs in clinical testing have either four or five SRs.
4. Choice and relative order of SRs
The various microdystrophin constructs in clinical testing with AAV vectors carry either four or five SRs.^29,30,43,50,83,85,86^ Since full-length dystrophin has 24 SRs, many combinations of SRs can be used to generate a protein expressed by a coding sequence small enough for packaging into AAV vectors.^87^ Most microdystrophin constructs published to date have retained the first and last SRs, as these SRs blend the central rod domain into the adjacent ABD and Dbds and are presumed to have a unique structure. Together these studies indicate that several combinations of four or five SRs can generate microdystrophins that are stable and that support normal muscle function.^30^

## RELATIONSHIP OF MICRODYSTROPHIN TO MUSCLE FUNCTION

Several clinical studies have quantified microdystrophin in muscle biopsies from patients who received intravascular AAV microdystrophin therapy. The expressed microdystrophin was appropriately membrane-localized and recruited members of the DAPC, demonstrating the clinical proof of concept for delivering functional microdystrophin. Interim results were presented from an ongoing Phase 1/2 study of a single infusion of SGT-001 (an investigational AAV9 microdystrophin gene therapy) at a dose of 5 × 10^13^ or 2 × 10^14^ vg/kg. In three patients receiving the higher dose, biopsies of skeletal muscle taken 3 months later showed widespread distribution of microdystrophin-positive muscle fibers with colocalization of neuronal nitric oxide synthase (nNOS) and β-sarcoglycan.^88^ Long-term biopsy data collected from these three patients, taken 2, 1.5, and 1 year after dosing, indicate evidence of durable and widespread expression of the microdystrophin.^89^

In addition, the North Star Ambulatory Assessment (NSAA) score was stable with minimal changes, and the 6-minute walk test (6MWT) distances were maintained at 1.5 years after treatment, suggesting clinical benefit compared with trajectories typically observed in natural history cohorts. Clinical data available at 24 months following infusion revealed continued patient benefit in maintaining motor function, as assessed by 6MWT and NSAA, when compared to natural history declines.^90^

One-year data from a Phase 1b study of an investigational AAV9 minidystrophin gene therapy (PF-06939926) showed that for the three patients in the low-dose cohort (1 × 10^14^ vg/kg), the mean proportion of muscle fibers expressing dystrophin was 28.5% at 2 months and 21.2% at 12 months after dosing.^91^ For the six patients in the high-dose cohort (3 × 10^14^ vg/kg), these measures were between 48.4% and 50.6% (*n* = 3), respectively. The patients also showed a functional improvement from baseline NSAA scores after 1 year compared with an external control group: an increase of one point for the study patients (*n* = 19) versus a median loss of four points for the external control group (*n* = 66; *p* < 0.005).^35^

Finally, in a Phase 1/2a study, four patients received a single dose of 2 × 10^14^ vg/kg recombinant AAVrh74 microdystrophin gene therapy. Transduction was confirmed in all patients, indicating successful delivery to skeletal muscle. At 12 weeks after treatment, 81.2% of muscle fibers expressed microdystrophin with a mean intensity of 96% at the sarcolemma. WB showed a mean expression of 74.3% without fat or fibrosis adjustment and 95.8% with adjustment. Microdystrophin expression also resulted in an increase in β-sarcoglycan, a component of the DAPC, suggesting that microdystrophin can promote restoration and reconstitution of the DAPC. In addition, functional outcomes (*e.g.*, NSAA score) were improved in these patients up to 1 year after treatment.^39^

Table 1 summarizes the AAV microdystrophin gene therapy clinical trials for patients with DMD listed on ClinicalTrials.gov that are recruiting; active, not recruiting; or enrolling by invitation.

**Table 1.** Adeno-associated virus microdystrophin gene therapy clinical studies from ClinicalTrials.gov (recruiting; active, not recruiting; enrolling by invitation)

ClinicalTrials.gov Identifier	NCT03375164	NCT03769116	NCT04626674	NCT05096221	NCT03362502	NCT04281485	NCT05429372	NCT03368742	NCT05693142
Company	Sarepta	Sarepta	Sarepta/Roche	Sarepta/Roche	Pfizer	Pfizer	Pfizer	Solid Biosciences, Inc.	REGENXBIO Inc.
Phase	1/2	2	1	3	1	3	2	1/2	1/2
Gene Therapy	SRP-9001	SRP-9001	SRP-9001	SRP-9001	PF-06939926	PF-06939926	PF-06939926	SGT-001	RGX-202
Estimated Enrollment	4	41	46	126	23	99	10	16	18
Age	3 months to 7 years	4 to 7 years	3 years and older	4 to 7 years	4 years and older	4 to 7 years	2 to 3 years	4 to 17 years	4 to 11 years
Primary Endpoint	No. of participants with AEs	Change from baseline in quantity of microdystrophin protein expression as measured by western blot Change from baseline in NSAA total score	Change from baseline in quantity of microdystrophin protein expression at week 12, as measured by western blot	Change from baseline in NSAA total score at week 52	Incidence of dose- limiting safety or intolerability, as measured by treatment-related AEs	Change from baseline in NSAA	Incidence and severity of treatment-emergent AEs and serious AEs No. of participants with abnormal hematology test results No. of participants with abnormal biochemistry test results No. of participants with abnormal urine analysis No. of participants with abnormal and clinically relevant changes in neurological examinations No. of participants with abnormal and clinically relevant changes in body weight No. of participants with abnormal and clinically relevant changes in vital signs No. of participants with abnormal and clinically relevant changes on cardiac troponin I No. of participants with abnormal and clinically relevant changes on ECG No. of participants with abnormal and clinically relevant changes on echocardiography	Change from baseline in microdystrophin protein in muscle biopsies Incidence of AEs Incidence of clinical laboratory abnormalities Incidence of abnormalities in vital signs Incidence of abnormalities in physical examinations Incidence of abnormalities on ECGs	Incidence of AEs and serious AEs

AEs, adverse events; ECG, electrocardiogram; NSAA, North Star Ambulatory Assessment.

## QUANTITY AND DISTRIBUTION OF MICRODYSTROPHIN

Several factors must be considered when assessing microdystrophin expression, including quantity produced, distribution to skeletal muscles throughout the body, and distribution among muscle cells within a given muscle type. The exact quantity of dystrophin or microdystrophin required to improve clinical phenotype is not known. Levels of dystrophin in healthy humans vary widely, with differences of three- to fivefold observed across sampled levels.^92,93^ In addition, some deletion mutations, such as exons 3–7 or 3–9 deletion, can lead to a low-level expression of shortened dystrophin and a milder phenotype, implying that any increase in internally truncated dystrophin could be beneficial.^94–97^ Animal studies have also shown improvements in muscle function when dystrophin levels were increased from low levels.^98^ While not curative, these improvements might affect quality of life and stabilize disease progression, both of which would have clinical benefits for DMD patients.

In addition to the total quantity of expressed dystrophin or microdystrophin, the importance of the distribution of such expression is not fully understood. For example, the clinical benefit of 10% of fibers positive (on tissue section) with 50% total dystrophin (in whole-muscle lysate) versus 50% fibers positive (on tissue section) with 10% total dystrophin (in whole-muscle lysate) is not known. Studies in transgenic *mdx* mice expressing full-length dystrophin or microdystrophins, or in *mdx* mice injected with an AAV microdystrophin vector, have revealed that some protection from dystrophy occurs even if the expression is nonuniform, that is, mosaic.^29,47,49,99^ However, these studies also reveal that uniform expression is more protective than mosaic expression. Further, low-level expression of dystrophin in a uniform pattern tends to be more protective against necrosis than high-level mosaic expression.^47,98,100–104^

Another consideration is body-wide distribution. In nonclinical studies, multiple tissues can be evaluated to interrogate the distribution of expression. AAV-microdystrophin gene therapy treatment in animal models has been shown to result in robust body-wide protein expression. It is not feasible or ethical to perform muscle biopsies on multiple muscles to clinically assess systematic expression in DMD patients. Preclinical data have shown the presence of microdystrophin in skeletal and cardiac muscles after administration of AAV microdystrophin gene therapy in both dystrophic dogs and mice, confirming body-wide expression. Data from dystrophic dogs show protein expression in both skeletal and cardiac muscle.^38,61,62^ Data from dystrophic mouse models reinforce the findings of widespread protein expression seen in dystrophic dogs.^36,50,52,54,57,58,86,105^ Similarly, skeletal and cardiac expression was seen with administration of AAV reporter gene in a dystrophic dog model.^38^

## BIOPSY AND MEASUREMENT METHODS

A muscle biopsy is required to quantify dystrophin and microdystrophin levels in DMD patients. These biopsy and assay methods have been used not only in clinical practice but also as the basis for regulatory approval. The techniques for obtaining, freezing, and handling samples to ensure that the tissue is appropriate for analysis have been well documented.^106,107^ Techniques have improved in recent years, limiting the amount of tissue needed to reduce the burden on patients.

Multiple techniques, beyond the scope of this article, have been developed to characterize and quantify dystrophin and microdystrophin. These include WB, capillary-based WB, mass spectrometry (MS), and immunofluorescence staining (IF), and they each provide critical, yet, limited information about the presence of dystrophin or the transduced microdystrophin. Collectively, they can provide complementary evidence of the appropriate localization of microdystrophin (IF), protein integrity and relative quantitation (WB, capillary-based WB), and absolute amount (MS).^93,108–112^ All validated techniques have demonstrated dystrophin or microdystrophin expression, dose response, and sarcolemmal localization.

## Conclusions for Appropriate Use of Microdystrophin as a Surrogate Endpoint

The use of microdystrophin as a reasonably likely surrogate endpoint is supported by the evidence provided. The use of microdystrophin is based on the biological relationship and the primary defect of DMD patients, which is the lack of dystrophin. Microdystrophin is rationally designed to incorporate critical elements of a full-length dystrophin protein so that it is functional, correctly localized to the muscle cell membrane, recruiting members of the DAPC, and stabilizing the muscle. Based on preclinical and clinical data, microdystrophin can be evaluated as a surrogate endpoint as early as 2 months after dosing to support accelerated approval in clinical trials. However, current microdystrophin clinical trials are enrolling few nonambulatory patients, and no patients older than 17 years of age.

Consequently, it is difficult to predict functional outcomes that might result from treatment of adult patients. Studies in old mdx mouse models for DMD have shown a benefit in both skeletal and cardiac muscles.^57,58,113^ Also, microdystrophin expression halts ongoing myofiber necrosis, suggesting that at a minimum loss of muscle mass and function might be preserved in microdystrophin treated patients. In this case measurements of microdystrophin expression could prove more informative than attempts to measure clear increases in strength or other functional outcomes in older patients.

It is important to acknowledge the importance of safety data for approval also. FDA has noted that “…what is a feasible and sufficient safety assessment is a matter of scientific and regulatory judgment based on the particular challenges posed by each drug and disease, including patients' tolerance for risk in the setting of unmet medical need.”^114^ With respect to rare diseases, the FDA has explained that “…[t]he goal of safety evaluation during drug development is to characterize the drug's safety profile in a reasonable number of patients over a reasonable duration of time, consistent with the intended use of the drug.”^114^ For rare diseases, however, “reasonable” requires “…consideration of feasibility challenges posed by the limited number of patients with the disease.”^114^

FDA has also explicitly said that when considering the benefit-risk framework and making regulatory decisions regarding drugs and biologics for dystrophinopathies, it will “…consider patient and caregiver tolerance for risk and the serious and life-threatening nature of these conditions. For example, patients may be willing to tolerate substantial risk of harm if a drug might delay loss of important abilities such as ambulation. However, tolerance for risk may vary among individuals and be affected by disease stage and severity; FDA would consider this heterogeneity in regulatory decisions.”^9^

Gene therapies approved using the accelerated approval pathway can leverage long-term follow-up (LTFU) studies—required to identify and mitigate long-term risks associated with the therapy—to also verify and describe the predicted clinical benefit. As discussed in FDA's guidance on this topic, the “…LTFU protocol for gene therapy trials is primarily designed to capture delayed adverse events in study subjects as well as to understand the persistence of the GT product. As a sponsor, you may consider designing the LTFU protocol to assess the long-term clinical efficacy and durability of your product.”^115^ This approach will allow efficient assessment of patients for both safety and efficacy and help ensure timely completion of postapproval studies required under accelerated approval.
